# The New Woman and the “Bicycle Craze”

**Published:** 1895-09

**Authors:** 


### The New Woman and the "Bicycle Craze."

The medical journals and the secular press with the comic squib-maker and cartoonist are just now reviewing the new woman with her bloomers and bicycle. The result is some sensible suggestions and a great deal of nonsense.

Whether the bicycle craze will finally be promotive of good to the race is just now a mooted question. Much may be said wisely for and against the pastime. It must be delightful for any one who is master of his equilibrium, on two wheels a-tandem, to take before sunrise a whirl of five or ten miles upon the lane, pike, or boulevard. And indeed such manly (womanly?) exercise ought to be healthful; but when we see men and women wearily working their way among various vehicles of a dusty or muddy business street, or hear them boast of riding fifteen or twenty miles without a stop, we may be pardoned a reasonable doubt if good be so secured. Intemperance in physical sport may be as much a menace to the full development of the coming man and woman as the intemperance of sloth, appetite, and passion.

To say nothing of the accidental features of wheeling, which are at least too numerous, there is much in the stooped position of the rider, the peculiar strain upon the great lumbar and pelvic muscles, and the pressure exerted upon the tuber ischii, and sometimes perineum, to cause the hygienist to think several times before he gives the sport his unqualified approval.

There is no question that urethral troubles in the males are induced, or if previously existing, made worse by cycling. Nor has the assertion of the tendency of the sport to produce (by inducing kidney congestion) albuminuria been wholly set aside. Furthermore, in reference to the female, it may be seriously questioned if the position of the rider and pressure upon the tuber ischii are not likely to make dystocia more common in the woman of the future than it is or has been in the woman of to-day and of past generations.

But, these negative points aside, the bicycle has come to stay, and, whether its use be hygienic or otherwise, we must not tolerate it, but use all means in our power to make its use innocuous, if not promotive of health.

And in one great feature at least, the wheel seems likely to become a blessing to womankind. This is in the matter of dress.

In a recent communication to the Record, Dr. W. H. F. Miller discusses this question to the point. Among other things he says:

"How often, when the icy blasts of winter have been blowing with hurricane force, have we seen female pedestrians clutching at their skirts to prevent them from being blown over their heads; and how often have we seen those same skirts inflated, not like a balloon with hot but with cold air. In fact, they are too often mere traps for collecting cold air. The pelvic organs are well supplied with blood-vessels, and it must follow that when the parts are suddenly chilled, the blood-supply is temporarily impaired. To say that such a thing is not harmful is of course absurd.

What is true of the full-grown woman is doubly true of her younger sister with shorter skirts. Now suppose the latest fad, bloomers, becomes the general dress of woman, all this will be done away with. The pelvic organs will be well protected from rapid changes of temperature. There will not be alternating congestion and anemia. There will be a general tone given to the pelvis which modern dress prohibits . . . .

We know that the higher we go in the scale of so-called civilization, the more neurotic the individual, and as society demands that its female members should do no work, so there follows a life of indolence, with lack of muscular development. The new woman is changing all this. She is engaging in outdoor exercise in very many different ways, and to enjoy this exercise, she has had to change her style of dress.

Neurasthenia is alarmingly prevalent among our women, and, for my part, I believe it is due as much to lack of protection to the pelvic organs from climatic change as to any thing else, and the next cause on the list is the lack of proper healthy exercise. A woman will sit in the house all day, and at nine o'clock at night go out to a dance. She will waltz until she is in a perspiration all over. She will be very solicitous about her shoulders, but will forget that the cool air will penetrate to the pelvis, so she throws a shawl over her shoulders and goes out into the night air to get cool. She does get cool, especially in the pelvic organs. The next day ovarian pains sets in, perhaps a cellulitis develops. She has a headache; the doctor comes and says, "You have caught cold," but no word of advice about avoiding cold to the pelvis. Neurasthenia supervenes. A young woman stands in a store all day; at night she is tired; she can not find enjoyment in walking. To hire a horse is beyond her means, so she sits down and reads; in a short time she becomes hysterical and neurotic.

All this can be remedied by following the lead of the new woman in her rational dress and exercise. Of course moderation must be exercised in all things.

The writer lays none too much stress upon the evils resultiing from the hygienically absurd manner in which our women dress, and if the bicycle shall secure to woman some rational means of protecting her lower limbs and delicate pelvic organs from the wind and weather, it will prove a lasting blessing to humanity.—*American Practitioner and News, July, '95.*

